# 伴有*CLIP1-ALK*融合基因晚期肺鳞癌1例

**DOI:** 10.3779/j.issn.1009-3419.2022.102.29

**Published:** 2022-09-20

**Authors:** 月 袁, 征 王, 鑫 聂, 萍 张, 琳 李

**Affiliations:** 1 100730 北京，国家老年医学中心，中国医学科学院老年医学研究所，北京医院肿瘤内科 Department of Oncology, Beijing Hospital; National Center of Gerontology, Institute of Geriatric Medicine, Chinese Academy of Medical Science, Beijing 100730, China; 2 100730 北京，国家老年医学中心，中国医学科学院老年医学研究所，北京医院病理科 Department of Pathology, Beijing Hospital; National Center of Gerontology, Institute of Geriatric Medicine, Chinese Academy of Medical Science, Beijing 100730, China; 3 100730 北京，中国医学科学院，北京协和医学院研究生院 Graduate School of Peking Union Medical College, Chinese Academy of Medical Sciences, Beijing 100730, China

**Keywords:** *ALK*融合基因, 肺肿瘤, ALK抑制剂, *ALK* fusion gene, Lung neoplasms, ALK inhibitor

## Abstract

间变性淋巴瘤激酶（anaplastic lymphoma kinase, *ALK*）融合基因是非小细胞肺癌的重要的肿瘤驱动基因，约占非小细胞肺癌患者的5%左右，其中97%为肺腺癌患者。自2007年首次在肺腺癌患者中发现棘皮动物微管相关蛋白样4（echinoderm microtubule-associated protein-like 4, *EML4*）-*ALK*融合以来，多种*ALK*融合伴侣相继被检测出来。本例晚期肺鳞癌患者通过二代测序（next generation sequencing, NGS）检测到*CLIP1-ALK*融合基因，并于2021年5月5日开始先后口服阿来替尼、恩沙替尼治疗，阿来替尼治疗有效，但于2021年9月30日去世。本文报道了接受ALK抑制剂治疗的*CLIP1-ALK*融合基因的肺鳞癌患者，并对其疗效进行讨论。

## Abstract (en)

Anaplastic lymphoma kinase (*ALK*) fusion gene is an important tumor driver gene of non-small cell lung cancer, accounting for about 5% of patients with non-small cell lung cancer, of which 97% are patients with lung adenocarcinoma. Since the first discovery of echinoderm microtubule associated protein-like 4 (*EML4*)-*ALK* fusion in patients with lung adenocarcinoma in 2007, a variety of *ALK* fusion partners have been detected. *CLIP1-ALK* fusion gene was detected by next generation sequencing (NGS) in this patient with advanced lung squamous cell carcinoma, and Alectinib and Ensartinib were taken orally on May 5, 2021. Aletinib was effective for this patient but the patients died on September 30, 2021. This is a report of lung squamous cell carcinoma patients with *CLIP1-ALK* fusion gene treated with ALK inhibitors.

## 1 病例资料

患者，男性，44岁，2021年3月因咳嗽、咳痰、咯血以及活动后喘憋就诊于外院，行胸部计算机断层扫描（computed tomopraphy, CT）提示左肺占位，后行支气管镜下肺活检，病理结果回报：鳞癌，低分化。2021年4月14日行正电子发射计算机断层显像（positron emission tomography/CT, PET/CT）：①左肺门软组织密度肿块，伴高代谢，符合肺癌表现；②左肺门、纵隔及左锁骨上区淋巴结多发转移；③左侧胸膜转移，伴大量胸腔积液；④胸骨及左侧前锯肌转移。个人史：吸烟10余年，平均10支/日。该病例报道已获得患者家属知情同意。患者入我院时咳嗽、咳痰及活动后喘憋症状明显，影响日常活动。完善肿瘤标志物检测：癌胚抗原（carcinoembryonic antigen, CEA）11.2 ng/mL，鳞状上皮细胞癌抗原（squamous cell carcinoma antigen, SCC-Ag） > 70.0 ng/mL。予患者行胸腔引流管植入术后完善胸腔积液病理：（胸腔积液）找到癌细胞，考虑为低分化鳞癌；免疫组化结果：细胞角蛋白7（cytokeratin 7, CK7）（+++），细胞角蛋白20（cytokeratin 20, CK20）（-），甲状腺转录因子1（thyroid transcription factor-1, TTF-1）（-），天冬氨酸蛋白酶A（novel aspartase proteinase A, Napsin A）（-），CDX2（-），P40（++）（图 1A），CK5/6（+++），P53（70%+特殊染色），CEA（极个别肿瘤细胞+），Ki67（40%+），glut1（+++），PAX8（-），D-PAS（-），Syn（-），CgA（-），Ventana ALK（D5F3）（+）（图 1B），ALK（对照）：（-）。

**图 1:**
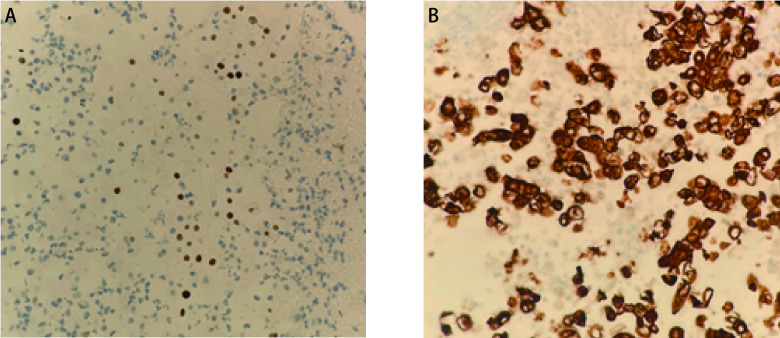
患者胸腔积液细胞免疫组化染色结果。A：P40免疫组化染色结果（×400）；B：Ventana ALK（D5F3）染色结果（×400）。 Immunohistochemical staining results of pleural effusion cell block. A: P40 immunohistochemical staining results (×400); B: Ventana ALK (D5F3) immunohistochemical staining results (×400).

取胸腔积液进一步完善分子分型相关检测：首先完成肺癌常见9项驱动基因突变阻滞扩增系统（amplification refractory mutation system, ARMS）检测，结果显示*EML4-ALK*融合基因：阴性（试剂购于厦门艾德生物医药科技股份有限公司）。进一步完善*ALK*基因融合荧光原位杂交（fluorescence *in situ* hybridization, FISH）结果：阳性（试剂购于武汉康录生物技术股份有限公司）（图 2）。随后进行40基因NGS（RNA based-扩增子建库NGS，检测平台：Illumina Nextseq CN500）：*ALK*（Fusion, SNV, InDel）：未检出。为了明确该患者*ALK*融合伴侣情况，我们又进行了胸腔积液1021基因NGS（DNA based-靶向捕获建库NGS），结果显示*CLIP1-ALK*融合（融合功能区域：EX10:EX20），突变频率0.9%；肿瘤突变负荷（tumor mutational burden, TMB）0.63 Muts/Mb，MSS（检测平台：Illumina HiSeq 2000/2500）。程序性死亡配体1（programmed death ligand 1, PD-L1）免疫组织化学染色（22C3）结果：表达肿瘤细胞阳性比例分数（tumor proportion score,  TPS）5%、综合阳性评分（combined positive score, CPS）5。

**图 2:**
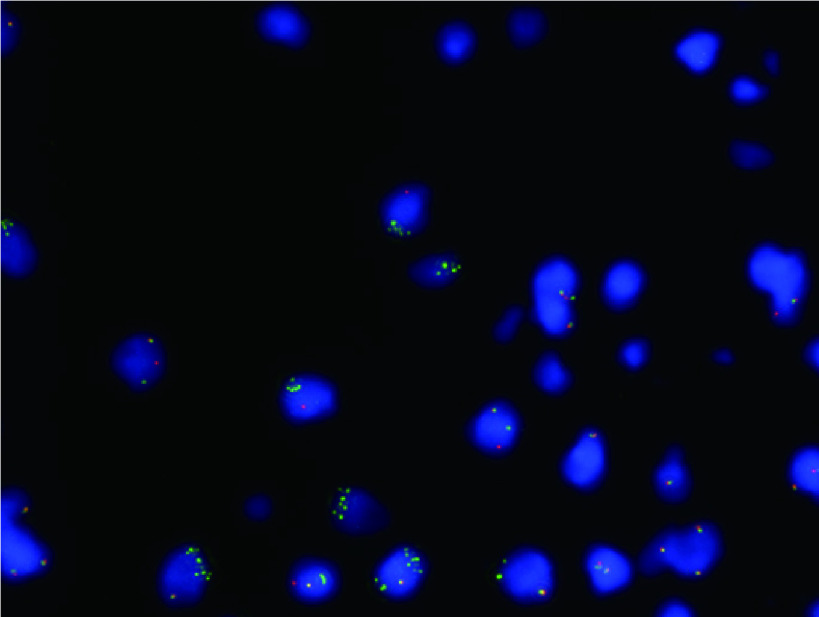
患者胸腔积液ALK荧光原位杂交结果 Results of ALK fluorescence *in situ* hybridization in pleural effusion. ALK: anaplastic lymphoma kinase.

结合临床症状及辅助检查结果，该患者诊断为晚期肺鳞癌Ⅳ期，*ALK*驱动基因阳性，体能状态评分（performance status, PS）2分，完善基线影像学评估（图 3A）后于2021年5月5日起给予阿来替尼600 mg，*bid*，口服，作为一线治疗，患者用药10 d后咳嗽喘憋症状较前缓解，2021年7月14日复查胸部CT：左肺肿块范围较前缩小，左上肺较前复张（图 3B），复查SCC-Ag 14.6 ng/mL，根据实体瘤疗效评价标准（Response Evaluation Criteria in Solid Tumors, RECIST）1.1评价疗效：病情稳定（stable disease, SD），同时患者喘憋症状明显改善，活动耐量较前增加，此时PS评分为1分，继续给予阿来替尼治疗。2021年9月患者受凉后出现呼吸道感染，临床症状再次加重，一般情况明显恶化，2021年9月15日CT示：左肺肿块明显增大，右肺转移瘤及纵隔各区淋巴结较前增大，胸腔积液及心包积液较前增多（图 3C），复查SCC再次升高：SCC＞70.0 ng/mL，RECIST 1.1评价疗效：疾病进展（progressive disease, PD）。2021年9月17日起采用恩沙替尼225 mg，*qd*，口服，患者咳嗽症状持续加重，积极对症处理无效，患者于2021年9月30日因PD去世。

**图 3:**
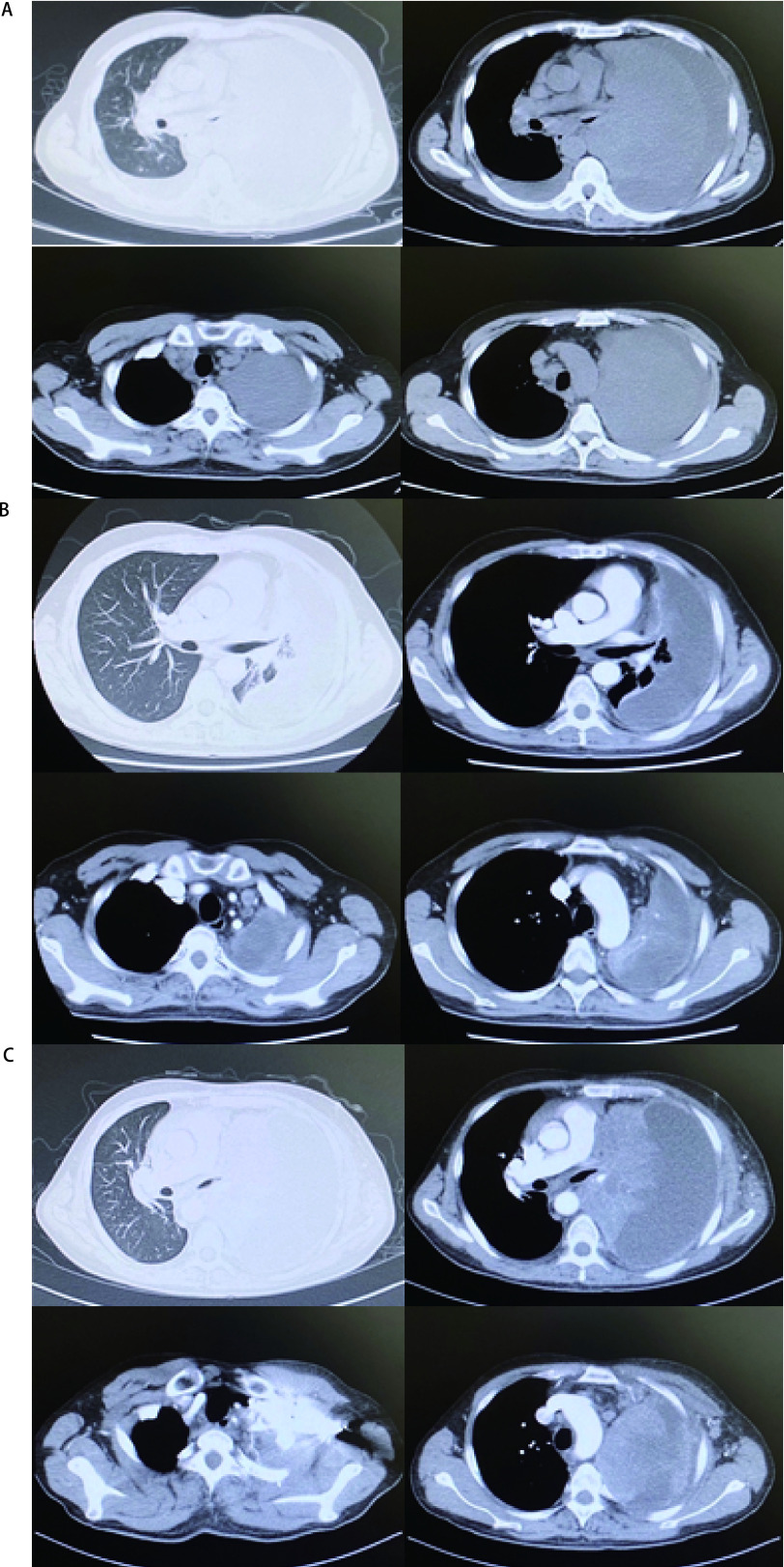
胸部CT对比。A：患者基线影像学检查（2021年4月26日）：可见左主支气管截断，左肺致密实变影（肿瘤病灶与肺不张分界不清），纵隔多发淋巴结肿大；B：2021年7月14日疗效评价：左肺肿块与左上肺分界不清，范围较前缩小，左上肺较前复张；纵隔及右侧肺门多发肿大淋巴结较前缩小；C：患者2021年9月15日疗效评价：左肺肿块，较前范围明显增大，伴左侧全肺不张，纵隔6区淋巴结较前明显增大。 Chest CT contrast. A: Baseline imaging examination of patient (April 26, 2021): truncation of the left main bronchus, dense consolidation of the left lung (unclear boundary between tumor and atelectasis) and multiple mediastinal lymph gland can be seen; B: Effect evaluation on July 14, 2021: the boundary between the left lung mass and the left upper lung is unclear, the scope is narrower than before, and the left upper lung is more dilated than before; the mediastinum and the right hilum of the lung were multiple and the lymph nodes were smaller than before; C: Effect of evaluation on September 15, 2021: the left lung mass is significantly larger than before, accompanied by left total atelectasis, and para-aortic lymph nodes was significantly larger than before. CT: computed tomography.

## 2 讨论

本文报道了1例*ALK*阳性晚期肺鳞癌患者，免疫组织化学及FISH检测*ALK*阳性，NGS检测出*CLIP1-ALK*融合基因，一线使用阿来替尼有效，一线治疗无进展生存期（progression-free survival, PFS）为4.5个月。总生存期（overall survival, OS）为6.0个月。从病理分型角度来说，低分化肺鳞癌和肺腺癌组织通过常规光学显微镜较难区分，特别是在小活检和细胞学样本中，诊断依赖免疫组化生物标志物^[4]^，本例患者免疫组化显示P40（++），CK5/6（+++），CK7（+++），TTF-1（-），Napsin A（-），根据国际肺癌研究协会/美国胸科协会/欧洲呼吸协会（International Association for the Study of Lung Cancer/American Thoracic Society/European Respiratory Society, IASLC/ATS/ERS）分类标准考虑低分化鳞癌^[5]^。相比于肺腺癌，*ALK*融合基因在肺鳞癌中较为罕见，发生率为1%-2.5%^[6, 7]^。*ALK*阳性肺鳞癌患者使用ALK抑制剂效果相较于肺腺癌患者较差。Meng等^[8]^回顾了单中心个案报道中共31例*ALK*阳性肺鳞癌患者，共有20例患者接受了ALK抑制剂作为一线或二线治疗，治疗的中位持续时间为（6.4±4.4）个月，远低于ALK抑制剂在*ALK*阳性肺腺癌中取得的疗效。Lewis等^[9]^回顾了6例具有*EML4-ALK*重排的肺鳞癌患者的治疗过程，使用ALK抑制剂作为一线或二线治疗的中位PFS为2.8（1.8-6.3）个月，OS为8.3（3.2-32.1）个月。本文中患者OS与既往文献^[9]^报道相近。

目前获2019年中国NSCLC ALK检测专家共识推荐用于*ALK*基因融合检测手段有FISH、实时荧光定量聚合酶链反应（real-time fluorescence polymerase chain reaction, RT-PCR）、免疫组织化学（immunohistochemistry, IHC）及NGS等方法^[10]^。美国国立综合癌症网络（National Comprehensive Cancer Network, NCCN）NSCLC临床实践指南中推荐使用获美国食品药品监督管理局（Food and Drug Administration, FDA）批准的免疫组织化学Ventana-D5F3作为独立检验使用，无需FISH确认^[11]^。本例患者免疫组织化学及FISH检测阳性，但由于RT-PCR只能针对已知*ALK*融合基因类型、扩增子测序（amplicon-based NGS）方法局限于特定的常见位点，上述两种检测方法结果阴性，最终通过大panel NGS检出罕见融合伴侣，提示对于罕见融合基因类型来说，IHC Vatana-D5F3 ALK及NGS有其独特应用价值。

*ALK*基因的易位导致ALK融合蛋白的产生，这些融合蛋白发生二聚化以激活ALK下游信号通路，在血液肿瘤及实体瘤中发挥致癌驱动作用^[12]^。目前*ALK*阳性NSCLC中，*EML4-ALK*是主要融合变体，约占95%^[13]^。随着高通量测序技术及靶向RNA测序技术在*ALK*阳性NSCLC患者中的应用，越来越多的罕见融合伴侣被检测出来。截止2020年1月，已在NSCLC患者中发现至少90个不同的ALK融合蛋白^[14]^。ALK融合蛋白转录的起始是由伴侣基因的调控区驱动的，其亚细胞定位是由伴侣蛋白决定的；ALK融合体通过ALK伴侣蛋白发生二聚化及反式自磷酸化，从而激活ALK激酶结构域^[12]^。一些回顾性研究^[15-17]^提示携带不同*EML4-ALK*变体的患者使用ALK抑制剂疗效不同，已有体外实验^[18]^证明，ALK融合伴侣的不同可影响肿瘤细胞的细胞表型、生化特性及对ALK抑制剂的反应性。提示携带不同伴侣蛋白的*ALK*阳性NSCLC为一组异质性疾病。

本文中患者通过胸腔积液高通量测序检测出*CLP1-ALK*融合基因，含CAP-Gly结构域的细胞质连接蛋白-1（CAP-gly domain containing linker protein 1, CLIP1）是细胞骨架相关蛋白家族的成员，具有保守的富含甘氨酸的结构域，它与微管结合，在细胞内囊泡运输中发挥重要作用^[19]^。这一类型的*ALK*重排首次发现于Spitz肿瘤中，Yeh等^[20]^于2015年报道了具有*CLIP1-ALK*融合的Spitz肿瘤，融合断点位于*CLIP1*外显子13和*ALK*外显子20。2017年，Vendrell及其同事首次报道了NSCLC中的*CLP1-ALK*（C22:A20）融合基因，融合断点位于*CLIP1*外显子22和*ALK*外显子20，该例患者对克唑替尼治疗有反应^[21]^。2019年，Pinsolle等^[22]^报道了1例具有*CLIP1-ALK*融合且具有神经内分泌特征的NSCLC患者（71岁女性），融合断点位于*CLIP1*外显子12和*ALK*外显子20，该例患者在开启治疗之前因PD死亡。本文报道了1例接受ALK抑制剂治疗的*CLP1-ALK*融合患者，并且与之前文献报道中具有不同的融合断点，该患者使用ALK抑制剂治疗效果不佳，阿来替尼作为一线治疗PFS为4.5个月，对比ALEX研究（阿来替尼对比克唑替尼一线治疗Ⅲ期临床研究）中阿来替尼治疗组PFS为34.8个月^[23]^，提示*CLP1-ALK*融合基因NSCLC恶性程度高，对ALK抑制剂反应较差。

研究^[24]^显示，*CLIP1-LTK*融合是新的NSCLC驱动基因。白细胞酪氨酸激酶（leukocyte tyrosine kinase, LTK）和ALK构成受体酪氨酸激酶的ALK/LTK亚家族，它们连同其激活细胞因子ALKAL1和ALKAL2，参与调控神经发育、癌症和自身免疫性疾病，LTK及ALK各自的激酶结构域中具有近80%的同一性^[25]^。考虑到上述因素，Izumi等^[24]^在细胞实验中证实ALK抑制剂可抑制CLIP1-LTK激酶活性并诱导肿瘤细胞凋亡，劳拉替尼效果最好，其他ALK抑制剂（克唑替尼、色瑞替尼、阿来替尼、恩沙替尼、布加替尼）次之且效果相似，研究者在1例携带*CLIP1-LTK*融合基因的NSCLC患者（一线免疫单药联合化疗治疗后进展）中进行试验性劳拉替尼治疗，该患者表现出良好的临床反应，治疗5个月后肿瘤持续缩小。相比于该例*CLIP1-LTK*融合患者，本文中患者使用二代ALK抑制剂效果不佳，提示*CLIP1-LTK*融合和*CLIP1-ALK*融合不尽相同，劳拉替尼能否在*CLIP1-ALK*融合患者中收获较好疗效有待进一步研究证实。

本文报道了首例接受ALK抑制剂治疗的罕见*CLIP1-ALK*融合基因的晚期肺鳞癌患者，该例患者使用阿来替尼治疗有效但总体效果差，OS短。
